# Changes in child exposure to secondhand smoke after implementation of smoke-free legislation in Wales: a repeated cross-sectional study

**DOI:** 10.1186/1471-2458-9-430

**Published:** 2009-11-24

**Authors:** Jo C Holliday, Graham F Moore, Laurence AR Moore

**Affiliations:** 1 Cardiff Institute of Society, Health and Ethics, School of Social Sciences, Cardiff University, 1-3 Museum Place, Cardiff, CF10 3BD, UK

## Abstract

**Background:**

Smoke-free legislation was introduced in Wales in April 2007. In response to concerns regarding potential displacement of smoking into the home following legislation, this study assessed changes in secondhand smoke (SHS) exposure amongst non-smoking children.

**Methods:**

Approximately 1,750 year 6 (aged 10-11) children from 75 Welsh primary schools were included in cross-sectional surveys immediately pre-legislation and one year later. Participants completed self-report questionnaires and provided saliva samples for cotinine assay. Regression analyses assessed the impact of legislation on children's SHS exposure at the population level, and amongst subgroups defined by parental figures who smoke within the home.

**Results:**

Geometric mean salivary cotinine concentrations were 0.17 ng/ml (95% CI 0.15,0.20) pre-legislation and 0.15 ng/ml (95% CI 0.13,0.17), post-legislation, although this change was not statistically significant. Significant movement was however observed from the middle (0.10-0.50 ng/ml) to lower tertile, though not from the higher end (>0.51 ng/ml) to the middle.

Reported exposure to SHS was greatest within the home. Home-based exposure did not change significantly post-legislation. Reported exposure in cafés or restaurants, buses and trains, and indoor leisure facilities fell significantly.

The proportion of children reporting that parent figures smoked in the home declined (P = 0.03), with children with no parent figures who smoke in the home significantly more likely to provide saliva with cotinine concentrations of <0.10 ng/ml post-legislation.

Amongst children with no parent figures who smoke in the home, the likelihood of 'not knowing' or 'never' being in a place where people were smoking increased post-legislation.

**Conclusion:**

Smoke-free legislation in Wales did not increase SHS exposure in homes of children aged 10-11. Reported SHS exposure in public places fell significantly. The home remained the main source of children's SHS exposure. The legislation was associated with an unexpected reduction in cotinine levels among children with lower SHS exposure pre-legislation. The findings indicate positive rather than harmful effects of legislation on children's SHS exposure, but highlight the need for further action to protect those children most exposed to SHS.

## Background

An evolving body of literature demonstrates a multitude of deleterious associations with early lifecourse exposure to secondhand tobacco smoke (SHS: commonly known as 'passive smoke'), including increased risks of chronic obstructive pulmonary disorder, bronchiolitis and asthma [1-5] arising from underlying alterations in the development of lung structures [6]. Whilst the home is generally the main source of children's SHS exposure [7-10] and interventions to reduce children' s exposure have commonly focused upon exposure in the company of parents [11], the capacity for public health efforts to address children's exposure in the home may be limited to campaigns to promote voluntary restriction by adults [12,13], with legislation against smoking in the home being described as unacceptable in all but the most authoritarian of countries [14]. However, children may also be exposed to SHS in other contexts [8] such as hospitality establishments [15] and other public places [10].

Political and public support for legislative action to reduce SHS exposure in public places has grown rapidly in recent years. Following efforts of campaigners to heighten public awareness of the risks of passive smoking, engagement with opponents of legislation, and activities to win the backing of business [16], legislation prohibiting smoking in most public places has been introduced throughout the United Kingdom. Legislation came into effect in Scotland on March 26^th ^2006, in Wales on 2^nd ^April 2007, Northern Ireland on 30^th ^April 2007, and in England on 1^st ^July 2007, with the primary aim of protecting workers and the public from the harmful effects of SHS. A body of literature has begun to demonstrate some short term effects of smoking bans [17], with studies in Scotland and Ireland indicating that smoking bans have been effective in reducing SHS exposure in adults [18], particularly amongst barworkers [19,20]. Health benefits such as improved respiratory function [21], have also been observed.

Despite these positive impacts, concerns have been expressed regarding the potential displacement of smoking from public places into the home, affecting non-smokers and, in particular, children [14,22], although this proposition has found little support to date. Instead, increasing numbers of successful smoking cessation efforts amongst adults [23] and an increase in the proportion of smoke-free homes [24,25] have been observed. Hence, some commentators have speculated that smoke-free legislation may in fact reduce the prevalence of SHS exposure in the home [26].

Few studies have focused upon direct effects of smoke-free legislation on children's SHS exposure. The first such study, conducted in Scotland [7] found no evidence for displacement into the home. Furthermore, a 39% post-legislation decline in cotinine concentrations in non-smoking children was observed. Reductions were significant for children from smoke-free homes, and where only the father figure smoked, although no significant changes were observed for children with either two parent figures who smoked, or just a mother figure who smoked. Since legislation did not focus on reducing exposure amongst children, this alleviated concerns regarding displacement.

This paper reports results from the changes in child exposure to environmental tobacco smoke (CHETS) Wales study, part of the Welsh Assembly Government's commissioned research programme assessing the impacts of Welsh smoke-free legislation. CHETS Wales aimed to replicate the aforementioned CHETS study [7], using biochemical and self-report data to assess impacts of Welsh smoke-free legislation on children's SHS exposure. The aim of the current paper is to assess: population-level changes in salivary cotinine concentrations following the introduction of Welsh smoke-free legislation; children's perceived exposure to SHS pre- and post-legislation; and potential displacement of parental smoking amongst subgroups defined by the number of parent figures who smoke within the home.

## Methods

### Study design

CHETS Wales was a repeated cross-sectional study of year 6 (10-11 years old) school children in Wales. Data were collected immediately pre-ban (from 31^st ^January 2007 to 30^th ^March 2007) and one year after the initial data sweep (from 31^st ^January 2008 to 28^th ^April 2008).

### Sample

The study aimed to include students from a nationally representative sample of 80 Welsh primary schools. Based on the hypothesis that among children who live in households with other smokers, there will be no change in home-based SHS exposure post-legislation, the sample size was identified to have 80% power to demonstrate equivalence within 0.15 sd, assuming an intra cluster correlation (ICC) of 0.05, or within 0.2 sd with an ICC of 0.14. The same schools were approached pre-and post-legislation to minimise differences in the sample between survey years. The sampling frame, which included all state maintained schools in Wales, was stratified according to high or low free school meal entitlement (above or below population mean of 17.12%) and Local Education Authority. Within each stratum, schools were selected on a probability proportional to size (total number of students in the school). Where schools declined to participate, replacement schools were randomly identified from within the same stratum.

In each school, one year 6 (age 10-11) class was randomly selected to participate. Due to the small size of many Welsh primary schools, or due to requests from schools to include all of year 6, the whole of year 6 was often included in the survey. Some classes also included year 5 (i.e age 9-10), students who were taught alongside year 6 children. Pre- and post-legislation however, 97.6% and 97.4% of participating children were aged 10 or 11 years at the time of data collection.

### Consent

Consent was sought at three levels. First, a letter was sent to the headteacher of each selected school inviting them to participate. Schools were followed up by telephone if they did not respond. When schools consented, further details of the study were provided by telephone and arrangements for data collection made. Consent letters were then sent to parents/carers of all potential participants (including those in year 5) via the school. An 'opt out' system was adopted in most schools, whereby parents/carers were asked to inform the school if they *did not *wish their child to participate. Seven schools requested that an 'opt-in' consent procedure was used. A researcher contacted schools to ascertain numbers of eligible participants. At each data collection, students were given the opportunity to withdraw from the study.

### Data collection

Data were collected in the classroom by trained CHETS Wales staff. Class teachers were asked to be present, but not to intervene in the data collection unless asked to do so by the researcher. All students were asked to complete an anonymous questionnaire regarding their own smoking behaviour and that of friends and household members (including parental figures), smoking norms and attitudes, recent SHS exposure in a variety of public and private locations, and asthma. In order to enable comparability of results, most questionnaire items were identical to those used in CHETS [7]. Students also provided an anonymous saliva sample for cotinine assay using a cotton wool swab of a salivette^®^. Attempts were made to ensure that data collections in each school were conducted at a similar time of the week and day at each datasweep although this was not always possible due to other school commitments.

Anonymous saliva samples were linked to questionnaires by unique identification numbers. Absentees were followed up where absenteeism at data collection was greater than 40%. The study protocol and consent procedures employed were approved by the School of Social Sciences Ethics Committee, Cardiff University.

### Measures

#### Smoke-free legislation

The year of data collection (2007 or 2008) was used as a proxy for the primary independent variable, introduction of smoke-free legislation

#### Salivary cotinine concentrations

The study's primary outcome measure, salivary cotinine (a metabolite of nicotine), has been identified as the most reliable and suitable biomarker of exposure to tobacco smoke in the previous 72 hours [27-29]. Saliva samples were assayed using capillary gas chromatography with a specific nitrogen/phosphorus detector from a 100 μl sample [30] and which had a detection limit of 0.1 ng/ml. Assays were conducted by the laboratory used by CHETS, ensuring comparable results.

#### Smoking behaviour

Respondent smoking behaviour was measured using the HBSC scale [31]. Children who gave a response other than 'I do not smoke', or with salivary cotinine concentration above 15 ng/ml [32] were classified as smokers and excluded from analyses relating to cotinine.

#### Perceived frequency of SHS exposure

Children were asked to indicate how often they were in a place where people are smoking.

#### Locations of perceived SHS exposure

Children were asked if they had been in a car, a café, someone else's home, a bus or a train, an indoor leisure facility and the home on the previous day, and whether anyone was smoking there. They were also asked to indicate the amount of time spent there.

#### Parental smoking in the home

Parents were classified as smoking if the child reported that they 'smoke every day' or 'smokes sometimes'. Children were asked to identify whether parental figures (mother, father, stepfather or mother's partner, and stepmother or father's partner) smoked in the home. Children were subsequently categorised according to the number of parent figures who smoked within the child's home (neither parent figure, father figure only, mother figure only, or both).

#### Demographic covariates

Children were asked to indicate year and month of birth and age in years on the day of data collection was calculated (date of birth set to the 14^th ^of the month). Responses to items on the Family Affluence Scale (FAS: [33]), which includes measures of bedroom occupancy, car ownership, holidays and computer ownership, were summed and taken as a marker of socioeconomic status (SES). Measures of material affluence are typically completed more accurately by children than alternative markers of SES such as parental occupation or education [34]. The FAS has been validated favourably against other measures of SES such as parental occupation [33].

#### Time of day data collected

The time of data collection was included as a control variable in adjusted regression analyses. Times were divided into three categories (9-11 am, 11 am-1 pm and 1 pm to 3 pm).

### Statistical analysis

Analyses replicated those of Akhtar et al [7]. However, compared to the Scottish dataset, the Welsh dataset had a higher degree of positive skew, with 47% of samples containing cotinine concentrations below the limit of detection. Hence, a number of supplementary analyses were included. All statistical analyses were conducted using the svy settings of Stata version 9, to adjust for the clustered nature of the data sample.

### Population change in secondhand smoke exposure

Saliva samples containing cotinine concentrations below the limit of detection (0.10 ng/ml) were assigned a random value from the left tail of a truncated log-normal distribution. Percentages of each sample whose cotinine concentrations were below 0.10 ng/ml or above noteworthy cutpoints [7,35] were calculated.

Median cotinine concentrations pre- and post-legislation were presented alongside modelled estimates of geometric mean concentrations (both unadjusted and adjusted for age, family affluence and time of day data collected) for each survey year. To adjust for the time of day saliva samples were collected, dummy variables were used for 11 am-1 pm collections and 1 pm-3 pm collections, with 9-11 am collections acting as the reference category. Modelled estimates, and significance of change, were assessed using linear regression models, with log-transformed cotinine values as the dependent variable. Since linear analyses were compromised by reliance on imputation for 47% of cases, the distribution was split into approximately equal tertiles ('low' <0.10 ng/ml, 'medium' = 0.10-0.50 ng/ml, 'high' >0.50 ng/ml) and analysed using multinomial logistic regression.

### Perceived exposure to secondhand smoke

Frequencies and percentages of children reporting each category of perceived frequency of SHS exposure pre- and post-legislation were calculated. Significance of change over time was assessed using multinomial logistic regression analysis.

Frequencies and percentage responses to items relating to locations of self reported exposure to SHS were calculated and significance of change over time assessed using design adjusted chi-squared analyses, comparing the proportion of children reporting that someone was smoking in the location in question against all other responses (including missing values).

### Displacement of parental smoking

Sub-group analyses examined cotinine concentrations and perceived frequency of SHS exposure, pre- and post-legislation, by number of parent figures who smoked within the home (no parent figures, father figure only, mother figure only, or both). One hundred and twelve children pre-legislation and 122 post-legislation responded only to items relating to a mother figure or a father figure, and most reported that this parent smoked in the home. Thus it was assumed that this question had been interpreted as requiring a response only if the parent smoked in the home and these children were classified as having one parent who smoked in the home.

Frequencies and percentages of children in pre- and post-legislation samples within each subgroup were calculated and significance of change in percentages of children with parents who smoke in the home were assessed using design adjusted chi-squared analyses. Finally, the linear and multinomial regression analyses described above were repeated for each subgroup.

## Results

### Response rates

Of the original 80 schools identified, 30 declined to participate in the study, and one did not respond. Thirty nine schools were identified to replace these schools, of which 27 agreed. Of the remaining 12, ten declined to participate and two did not respond. Schools which did not respond were not replaced due to time constraints. Seventy-six schools agreed to participate and one subsequently dropped out prior to the pre-legislation survey. A range of state-maintained schools participated, including Welsh medium (n = 13), bilingual (n = 9), and denominational schools (n = 17). The number of year 6 students ranged from 6 to 104 per school (mean 37) pre-legislation and from 7 to 119 (mean 37) post-legislation. Proportions of students entitled to free school meals ranged from 1.0% to 70.8% (mean entitlement 18.9%); similar to the Welsh national average of 17.1% (range 0%-78.6%). The proportion of schools with a free school meal entitlement above the national average was 44%, compared to 39.8% nationally.

Pre-legislation, opt-in consent was received for 117 (68.4%) children and 102 (5.8%) children were opted out. Post-legislation, opt-in consent was received for 85 (51.2%) children and 90 (5.1%) children were opted out. The mean number of students per class selected to be involved in each school was 25 pre-legislation (range 5-51) and 26 post-legislation (range 7-77). Pre-legislation, 1611/1761 (91.5%) students within 75 schools completed the smoking questionnaire. Post-legislation, 1605/1775 (90.4%) students completed the smoking questionnaire. Saliva cotinine concentrations were obtained for 1526 children (86.7%) in 75 schools pre-legislation (79 refused, 137 were absent and 19 were of insufficient sample volume/were contaminated) and for 1503 children (84.7%) within 71 schools post legislation (53 refused, 177 were absent and 42 were of insufficient sample volume/were contaminated).

For analyses relating to cotinine, only the 71 schools in which cotinine data were available at both time points were included, comprising 1447 children pre-legislation (82.2% eligible) and 1461 children post-legislation (82.3% eligible). In these schools, there was no significant difference between survey years in the day of the week of survey (p = 0.75), but 12 data collections (11.6% pupils) were conducted after 13.00 hrs pre-legislation compared to 19 (22.6% pupils) post-legislation (p = 0.09). Other analyses (i.e. using questionnaire data but not cotinine) used data gathered in 75 schools.

### Sample characteristics

Characteristics of pre- and post-legislation samples are presented in Table 1. Independent samples t-tests (for age) and design adjusted chi-squared analyses (for all other characteristics) indicated no significant differences between survey years.

**Table 1:** Description of sample pre- and post- Welsh legislation

Characteristic	2007(n = 1611) n (%)	2008(n = 1605) n (%)
Mean age* (years)	10.97(.41)	10.94(.49)
Boys	778(49.53)	792(49.38)
Family affluence scale**	(n = 1555)	(n = 1528)
Low	422(27.14)	360(23.56)
Medium	606(38.97)	621(40.64)
High	527(33.89)	547(35.80)
Self-reported smoking status		
Non-smokers	1569(97.39)	1580(98.44)
Smokers	24(1.49)	18(1.12)
Missing	18(1.12)	7(0.44)
Cotinine confirmed smoking status***		
Non-smokers	1405(97.09)	1434 (98.15)
Smokers****	26(1.80)	20(1.37)
Missing*****	16(1.11)	7 (0.48)
Family structure (parent figure that sample lives with)		
Both parents	1120(69.52)	1090(67.91)
Parent and step parent	159(9.87)	170(10.59)
Single mother	275(17.07)	266(16.57)
Single father	18(1.12)	24(1.50)
Grandparent	17(1.06)	20(1.25)
Other******	8(0.50)	10(0.60)
Missing	14(0.86)	25(1.56)

* age at date of data collection

** children who report living in both parent, step- or single parent families only (data broken into approximately equal tertiles, with differences in group sizes arising due to tied values: low = 4-8 on FAS scale, medium = 9&10, high = 11-13)

*** limited to children within 71 schools where children provided saliva samples at both time points

**** children classified as smokers if self report as smokers, or if cotinine concentration >15 ng/ml (regardless of self report)

***** students who did not answer the smoking question or who had a contaminated saliva sample, or insufficient sample volume

****** includes live in a foster home, or children's home, or that another adult lives with them

### Population change in secondhand smoke exposure

Almost half of saliva samples in both pre- and post-legislation samples contained cotinine concentrations below the limit of detection (0.10 ng/ml). Whilst there was no change in median cotinine concentrations, percentages of children above each cutpoint presented in Table 2 were slightly lower post-legislation. In particular, the percentage of children with cotinine concentrations below the limit of detection increased from 44% to 50% post-legislation, suggesting a greater degree of change for children with lower levels of SHS exposure pre-legislation.

**Table 2:** Cotinine concentration distribution and proportion of 10-11 year old children in Wales above each cut-point pre- and post-legislation

Measurement	2007(n = 1447)	2008(n = 1461)	p-value
Median cotinine concentration	0.10	0.10	-
Percentage below level of detection (0.1 ng/ml)	43.98	49.90	-
Percentage above cotinine concentration (ng/ml)			
0.1*	56.04	50.10	-
0.2	40.15	39.22	-
0.5	31.44	28.82	-
1	22.88	20.33	-
1.7	14.38	13.48	-
2	12.30	10.95	-
3	7.41	5.57	-
Unadjusted geometric mean (95% CI) cotinine concentration (ng/ml)**	0.17 (0.14 to 0.20)	0.15 (0.13 to 0.18)	0.10
Geometric mean (95% CI) cotinine concentration (ng/ml) adjusted for age, family affluence and time of day data collected**	0.17 (0.15 to 0.20)	0.15 (0.13 to 0.17)	0.07

*including 0.1

** non-smoking children only

Geometric means and 95% confidence intervals for cotinine concentrations at both timepoints among non-smoking children are presented in Table 2, and indicate an average 12% decline, from 0.17 ng/ml to 0.15 ng/ml. This change was not significant.

When the data were divided into tertiles, a decrease from 30.4% to 28.2% was observed in the percentage of children with 'high' (>0.50 ng/ml) cotinine concentrations post-legislation. A decrease from 24.8% to 21.2% was observed in the 'medium' tertile (0.10-0.50 ng/ml), matched by a significant increase from 44.8% to 50.6% in the percentage of children with 'low' cotinine concentrations (see Table 3).

**Table 3:** RRRs* for the likelihood of 10-11 year old children in Wales providing 'low' or 'high'** cotinine concentrations post-legislation

	Low (<0.10 ng/ml)		High (>0.50 ng/ml)	
	RRR (95% CI)	p-value	RRR (95% CI)	p-value
**Whole sample unadjusted (n = 2839)**	1.32 (1.09 to 1.59)	<0.01	1.09 (0.90 to 1.31)	0.38
**Whole sample adjusted for age, FAS and time of day data collected** **(n = 2786)**	1.39 (1.14 o 1.69)	<0.01	1.11 (0.92 to 1.34)	0.28

* RRR = Relative risk ratios

** medium tertile set as base category

### Perceived exposure to tobacco smoke amongst children in Wales

The percentage of children reporting SHS exposure in cafés and restaurants, buses and trains and indoor leisure facilities (public locations) decreased significantly post-legislation (see Table 4). However, there was no significant change in the percentage of children reporting SHS exposure in the home, a car or someone else's home (private locations). Notably, small numbers of children reported exposure to SHS in public locations, with the home the most frequently cited source of SHS exposure at both time points.

**Table 4:** Exposure to secondhand smoke in private and public locations pre- and post-legislation amongst 10-11 year old children in Wales

Location	I wasn't in this location yesterday n(%)	No one was smoking there n(%)	Yes, someone was smoking there n(%)	I Don't know n(%)	Total n(%)
Home (P = 0.56*)					
2007	88(5.55)	1051(66.23)	328(20.67)	120(7.56)	1587
2008	64(4.05)	1071(67.78)	313(19.81)	132(8.35)	1580
Car (P = 0.98*)					
2007	383(24.55)	1011(64.81)	107(6.86)	59(3.78)	1560
2008	367(23.11)	1056(66.50)	107(6.74)	58(3.65)	1588
Someone else's home (P = 0.14*)					
2007	881(55.44)	454(28.57)	153(9.63)	101(6.36)	1589
2008	886(55.58)	496(31.12)	129(8.09)	83(5.21)	1594
Café or restaurant (P < 0.001*)					
2007	1363(86.16)	109(6.89)	40(2.53)	70(4.43)	1582
2008	1365(85.63)	151(9.47)	12(0.75)	66(4.14)	1594
Bus or train (P < 0.01*)					
2007	1352(85.52)	159(10.06)	16(1.01)	54(3.42)	1581
2008	1408(88.50)	129(8.11)	5(0.31)	49(3.08)	1591
Indoor leisure facility (P = 0.03*)					
2007	1200(75.95)	253(16.01)	38(2.41)	89(5.63)	1580
2008	1208(75.97)	294(18.49)	20(1.26)	68(4.28)	1590

* tests for changes between survey years based on number of children reporting someone smoking in a location versus all other responses (including missing); significance levels for design adjusted chi-squared analysis shown.

A decrease from 24.6% to 20.5% in children reporting exposure 'about every day', a decrease from 63.1% to 60.1% in those reporting exposure 'sometimes', an increase from 5.8% to 9.4% in those reporting 'never' being in a place where people are smoking, and an increase from 6.4% to 9.9% in those who 'did not know' how frequently they were in a place where people were smoking was observed. Children were significantly more likely to report 'never' being exposed to SHS, or not knowing how often they were exposed to SHS post-legislation relative to the likelihood of reporting exposure 'sometimes' (see Table 5). The percentage of children reporting exposure 'sometimes' and 'about every day' each fell by approximately the same amount over time, and it is likely that this accounts for the lack of change in the likelihood of reporting exposure 'about every day'.

**Table 5:** RRRs* for the likelihood of 10-11 year old children in Wales reporting perceived SHS exposure about every day or never** post-legislation

	I don't know		Never		About every day	
	RRR (95% CI)	p-value	RRR (95% CI)	p-value	RRR (95% CI)	p-value
**Whole sample unadjusted (n = 3201)**	1.62 (1.29 to 2.02)	<0.001	1.70 (1.34 to 2.17)	<0.001	0.87 (0.74 to 1.03)	0.11
**Whole sample adjusted for age and FAS (n = 3141)**	1.62 (1.28 to 2.05)	<0.001	1.67 (1.32 to 2.12)	<0.001	0.90 (0.75 to 1.06)	0.18

* relative risk ratios from design adjusted multinomial logistic regression models

** 'sometimes' set as base category

### Displacement of parental smoking

At both time points, just over half of all children reported that they did not have a parent figure who smoked (pre-legislation = 52.6%; post-legislation = 55.5%), with the remainder reporting that one (pre-legislation = 26.7%; post-legislation = 27.3%) or both (pre-legislation = 20.6%; post-legislation = 17.3%) parent figures smoked. Changes observed in the percentage of children who did not have a parent figure who smoked and the percentage of children reporting that both parents smoked were not significant (χ^2 ^= 1.75, P = 0.16).

Percentages of children reporting that neither parent figure smoked within the home increased from 63.2% to 66.8%, and the percentage of children reporting that both parents smoked in the home fell from 16.8% to 12.8%. There was little change in the percentage of children reporting that either only their mother (pre-legislation = 10.5%; post-legislation = 10.9%), or father (pre-legislation = 9.6%; post-legislation = 9.5%) smoked within the home. Differences were significant, indicating that post-legislation, significantly less children reported having parent figures who smoked within the home (χ^2 ^= 3.15, P = 0.03).

Differences in geometric mean cotinine concentrations between survey years were not significant for any subgroup defined by whether parents smoked in the home. The percentage of children assigned to the 'low' tertile of the cotinine distribution, was markedly higher for those with no parent figures who smoke in the home, than for those with one or both parent figures who smoke in the home (see Table 6). Multinomial logistic regression demonstrated that for those with no parent figures who smoke within the home, there was an increase from 64.4% to 70.0% (p < 0.01) in the percentage of children in the 'low' category (cotinine <0.10 ng/ml). There was also a 7% decrease in the percentage of children with 'low' cotinine concentrations amongst children whose father figure smoked within the home (p = 0.11). No notable changes were observed among children with mother figures who smoke in the home, or among those with two parent figures who smoke in the home. The percentages of children in the 'high' tertile were relatively stable over time for all subgroups. Whilst a significant increase in the relative likelihood of children with no parent figures who smoke in the home reporting 'high' cotinine concentrations was observed (p = 0.04), this was due to a 7% reduction in the base category (i.e. 'medium' tertile), rather than a substantial increase in the 'high' tertile.

**Table 6:** 10-11 year old children in Wales allocated to each tertile by number of parent figures who smoke in the home.

		Low (<0.10 ng/ml) n(%)	Medium (0.10-0.50 ng/ml) n(%)	High (>0.50 ng/ml) n(%)	Total n
**Neither parent smokes in home**	2007	549 (64.36)	244 (28.60)	60 (7.03)	853
	2008	638 (70.03)	198 (21.73)	75 (8.23)	911
**Father figure smokes in home**	2007	28 (20.59)	41 (30.15)	67 (49.26)	136
	2008	18 (13.64)	47 (35.61)	67 (50.76)	132
**Mother figure smokes in home**	2007	12 (8.39)	31 (21.68)	100 (69.93)	143
	2008	12 (9.22)	28 (19.86)	100 (70.92)	141
**Both parents smoke in home**	2007	8 (3.76)	19 (8.92)	186 (87.32)	213
	2008	7 (4.24)	15 (9.09)	143 (86.67)	165

Most children reported 'sometimes' being exposed to SHS, with relatively small percentages of each subgroup reporting that they are 'never' in a place where someone is smoking (see Table 7). Children who reported that their parents smoke in the home were more likely to report being exposed to SHS 'about every day' and less likely to report being 'never' or only 'sometimes' exposed to SHS. The percentage of children from each group not knowing how often they were in a place where people were smoking increased by 2-4%. Most groups also saw an increase in the percentage of children reporting 'never' being in a place where people were smoking. The only group for whom the percentage of children reporting SHS exposure 'about every day' changed markedly were those with two parent figures who smoke in the home.

**Table 7:** Frequency of SHS exposure pre- and post-legislation amongst 10-11 year old children in Wales by number of parents who smoke in the home

		I don't know n(%)	Never n(%)	Sometimes n(%)	About every day n(%)	Total n
**Neither parent smokes in home**	2007	84 (8.69)	79 (8.17)	693 (71.66)	111 (11.48)	967
	2008	120 (11.92)	128 (12.71)	666 (66.14)	93 (9.24)	1007
**Father figure smokes in home**	2007	6 (4.05)	5 (3.38)	87 (58.78)	50 (33.78)	148
	2008	10 (6.94)	4 (2.78)	82 (56.94)	48 (33.33)	144
**Mother figure smokes in home**	2007	5 (3.15)	2 (1.26)	80 (50.31)	72 (45.38)	159
	2008	12 (7.32)	4 (2.44)	70 (42.68)	78 (47.56)	164
**Both parents smoke in home**	2007	5 (1.96)	5 (1.96)	100 (39.22)	145 (56.86)	255
	2008	7 (3.61)	7 (3.61)	82 (42.27)	98 (50.52)	194

Multinomial logistic regression analyses indicated that amongst children with no parent figures who smoke in the home, the likelihood of not knowing (p = 0.01), or 'never' (p < 0.01) being in a place where someone smoked increased post-legislation. The relative likelihood of reporting being in a place where people smoke 'about every day' did not change significantly. No significant changes were observed for children with a mother, father, or two smoking parent figures who smoked in the home. When combined into a single group (one or both parents smoke within the home), there was an increased tendency for children not knowing whether they were in a place where someone smoked (p = 0.03).

## Discussion

### Main findings

CHETS Wales aimed to replicate the Scottish CHETS study [7]. Unlike the Scottish study, which demonstrated a 39% reduction in geometric mean cotinine concentrations post-legislation, this study demonstrated a non-significant 12% decline. When data were divided into tertiles, a significant movement towards the lower end of the distribution was observed, with the percentage of children with cotinine concentrations less than 0.1 ng/ml increasing from 45% to 51%. Hence, whilst an unintended outcome rather than an original aim of legislation, a lowering of exposure occurred. However, the lack of change at the top of the distribution indicates that effects were limited to children whose cotinine concentrations were relatively low pre-legislation.

Consistent with other research [7,8,10,15], the greatest self-reported prevalence of SHS exposure occurred within the home. Prevalence of exposure in the home, a car or someone else's home (private places) did not change significantly. By contrast, a reduction in SHS exposure in cafés or restaurants, buses and trains, and indoor leisure facilities (public places) post-legislation was observed. However, less than 3% of children reported being exposed to SHS in public places pre-legislation. Whilst statistically significant, the public health significance of these changes is likely to be limited compared to potential effects on adult SHS exposure.

Significant reduction in the frequency of perceived SHS exposure was observed post-legislation, with the percentage of children reporting being in a place where people were smoking 'about every day' falling by 4%, and a similar increase in the percentage of children reporting 'never' being in a place where people were smoking. Whilst the fact that young people still saw people smoking in public places may have implications for enforcement, it is possible that children did not distinguish between indoor and outdoor spaces in their responses, potentially overestimating exposure.

As in Scotland, there was no evidence of displacement of smoking from public places into the home. Exposure was greatest at both time points amongst children with two parent figures who smoked in the home, followed by those with a mother figure who smoked in the home, those with a father figure who smoked in the home, and lowest amongst those without parent figures who smoked in the home. However, linear analyses demonstrated no significant changes in geometric mean cotinine concentrations for any subgroup post-legislation. Categorical analyses demonstrated significant increases in the likelihood of children with no parent figures who smoked in the home providing a 'low' cotinine concentration post-legislation. However, as in linear analyses, no significant changes were observed for children with one or more parent figures who smoked in the home. These findings support population-level analyses which indicated greater beneficial impacts of smoke-free legislation post-legislation amongst children with relatively low baseline SHS exposure. A number of studies have assessed the health effects of SHS exposure amongst children. However, the quantification of SHS exposure using salivary cotinine measures is scarce [see, [36,37], for examples] and there is limited published evidence regarding the health effects of very low levels of SHS exposure. Whilst plasma cotinine concentrations of ≥ 0.17 ng/ml, have been linked to health outcomes such as endothelial dysfunction [35], the lower threshold at which SHS exposure begins to pose health risks is largely unknown. Given the small nature of declines and the fact that they occurred only at the bottom of the distribution, it is at present unclear what implications if any these observed changes may have for health outcomes in these children. However, the acknowledged susceptibility of children to the detrimental effects of SHS, particularly long-term exposure, supports the need to reduce home-based exposure amongst children who have parent figures who smoke in the home. This remains a challenge.

There was a small but significant decline in the percentage of children reporting having parent figures who smoked in the home, consistent with research which shows a tendency for smoke-free public places to stimulate reduction in smoking within the home [25]. It is possible that this decline is a result of increased success of cessation efforts following legislation, as reported elsewhere [23] or raised awareness of the dangers of passive smoking impacting on smoking within the home amongst smokers with children. The discrepancy between the non-significant change in reporting of exposure in the home on the previous day, and significant change in frequency of parental smoking in the home may be due to persons other than parent figures smoking in the home. In addition, analyses did not distinguish between parents who smoked in the home 'every day and those who smoked in the home 'sometimes', and it is possible that parents who stopped smoking in the home were those who had previously smoked relatively infrequently in the home.

Children whose parent figures did not smoke in the home were significantly more likely to report 'never' being in a place where people were smoking post-legislation. These children were also more likely to report not knowing how often they were in a place where somebody was smoking post-legislation. It is possible that reduced awareness of the frequency of SHS exposure may reflect a downward trend in exposure. Amongst all children with one or more parent figures who smoked in the home, there was a significant increase in the percentage of children reporting not knowing how often they were in a place where people smoked, suggesting that the lack of change in subgroups with one or both parent figures who smoked in the home may have been a result of diminished statistical power in these smaller groups.

It is possible that differences between the findings of the current study and those of the Scottish CHETS study [7] are due to floor effects associated with the lower pre-legislation salivary cotinine observed in Wales (median concentration = 0.10 ng/ml) compared to Scotland (median concentration = 0.30 ng/ml). The reasons for this difference are unknown but may be due to a general lower exposure in the population, sample coverage, timing of data collection or pre-legislation changes in Wales due to exposure to the extensive UK media coverage of the legislation in Scotland. Imputation of 47% of cotinine concentrations placed substantial limits on linear analyses compared to the Scottish study, in which only a quarter of cases were imputed.

### Strengths of the study

This study included a large nationally representative sample of state maintained primary schools, which together with high response rates from children at both data sweeps, ensures national generalisability of results. The study benefits from the use of salivary cotinine measures as a primary outcome; a method previously endorsed as a reliable indicator of exposure to secondhand smoke [27-29].

### Limitations of study

In addition to the aforementioned statistical limitations arising from the reliance upon imputation for 47% of children, which were overcome by supplementing linear analyses with multinomial logistic regression analyses, a clear limitation is the difficulty in firmly attributing change to legislation, due to the absence of a counterfactual. However, the maintenance of such a group would have been impracticable. A longitudinal study, following up children over time may have facilitated examination of change but would have made it impossible to distinguish between changes occurring due to increases in children's age, or due to legislation. Whilst the rationale for targeting this age group was to eliminate exposure to other SHS from, for example, peer smoking, generalisability is limited by focusing upon one year group.

Whilst there was relatively low uptake of the study by schools (63%), reducing the intended sample of 80 schools to 75, both the number of schools and students included exceeded the minimum required to achieve the intended power of 80%. Furthermore, the stratified random sampling procedure ensured a representative sample of schools were included. The suggestion that non-participation in CHETS may have been due to collection of saliva samples was not observed in CHETS Wales; only 5 (of 44) schools reported this as a concern. The main reason (n = 10) for non-participation was involvement in other health initiatives and research.

Finally, it is probable that self-reports underestimated actual exposure compared to cotinine measurements since cotinine measures reflect exposure over the previous few days, whilst self-report exposure reflected exposure on the previous day. Furthermore, whilst FAS is recognised as an appropriate self-report method for identifying different levels of affluence [33], we acknowledge that with evolving lifestyles, use of FAS to measure SES may involve a degree of misattribution.

## Conclusion

No evidence of displacement of parental smoking into the home was found. Indeed, there appeared to have been a small decline in the percentage of parents smoking within the home post-legislation, which may be a step towards these smokers quitting altogether. However, reductions in the percentage of children reporting that they had parents who smoke in the home did not appear to have translated into significant reduction in the prevalence of home-based SHS exposure, with the home remaining the main source of children's SHS exposure. The perceived prevalence of SHS exposure in public places fell. However, only small percentages of children reported SHS exposure in these locations pre-legislation. Changes in smoking in the home post-legislation are a positive step towards changing attitudes regarding the benefits of smoke-free environments. Thus, it will be important to maintain this momentum and encourage more parents to designate and maintain their homes and cars as smoke-free places.

This study demonstrates population-level reduction in exposure to SHS after the introduction of smoke-free legislation in Wales. However, reductions in cotinine concentrations were not sufficient to affect the overall group mean. Thus, exposure remains at a level of public health concern, with almost 40 percent of children having a cotinine concentration ≥ 0.17 ng/ml, a level associated with endothelial dysfunction [35], and almost 6 percent of children having salivary cotinine concentrations higher than those of non-smoking Scottish bar workers prior to the Scottish legislation (geometric mean 2.94 ng/ml; [20]). This suggests a need for direct action to reduce children's exposure to SHS in Wales. Whilst there was an increased likelihood of children providing a saliva sample with an undetectable level of cotinine, the percentages of children at greatest risk from SHS exposure remained relatively static. Furthermore, changes at the lower end of the distribution were significant only among children with parent figures who did not smoke in the home. Hence, the unintended benefits of the legislation were observed amongst those children who were least exposed to SHS pre-legislation. This underlines the need to reinforce to smokers with children that even modest SHS exposure can be harmful to the health of children.

The known association between SES and adult smoking rates [see, [38], for example], suggests that young people in lower socioeconomic groups may be more exposed to SHS in the home, and therefore least affected by smokefree legislation. Differential impacts by SES will form a focus of future analyses. Further research should examine the longer-term impact of smoke-free legislation in Wales. For example, whether downward trends in children reporting that parent's smoked within the home continue, and longer term impacts on the smoking behaviour of parents.

## Competing interests

The authors declare that they have no competing interests.

## Authors' contributions

JH was the study manager, led research design, co-ordinated the CHETS Wales survey, prepared study materials and led the preparation of drafts of the manuscript. GM performed statistical analysis, interpreted the data, and participated in preparation of drafts of the manuscript. LM was the study principal investigator, and advised on study design and data analysis. All authors read and approved the final manuscript.

## Pre-publication history

The pre-publication history for this paper can be accessed here:

http://www.biomedcentral.com/1471-2458/9/430/prepub
